# Measuring Sleep Quality Among Medical Students Using the Epworth Sleepiness Scale

**DOI:** 10.7759/cureus.63319

**Published:** 2024-06-27

**Authors:** Arjun Sharma, Joaquin Austerlitz, Fabian Najjar, James McDermott, Jacob Matalon, Madhu Varma

**Affiliations:** 1 School of Medicine, California University of Science and Medicine, Colton, USA

**Keywords:** epworth sleepiness scale (ess), medical students, sleep medicine, sleep quality, medical training

## Abstract

Introduction

While existing literature establishes the positive impact of sleep on test performance among medical students and its correlation with better outcomes among physicians, there is a notable gap in the quantitative understanding of how the transition from preclinical to clinical training affects sleep quality.

Methods

Our survey was sent to all medical students attending the California University of Science and Medicine between April 2023 and January 2024. The relative risks for having an Epworth sleepiness scale (ESS) greater than 10 were calculated and compared for various subgroups in our sample. Univariate logistic regression analysis was also carried out to assess the effect of covariates on our primary outcome.

Results

In total, our sample consisted of 124 medical students. Only 11.3% (n=14) were somewhat dissatisfied or very dissatisfied with their medical school experience. The relative risk of having an ESS > 10 when on clinical rotations was 2.06 (95% CI: 1.22-3.49).

Conclusion

This study demonstrates that the risk of medical students experiencing excessive sleepiness, defined by an ESS > 10, doubles when students are on clinical rotations. Despite being limited by information bias and a smaller sample size, this study provides interesting pilot data on the quantitative examination of sleepiness among medical students and may be used to guide areas for future work.

## Introduction

Medical students frequently endure poor sleep quality, a consequence of their demanding schedules, characterized by intensive studying and clinical/non-clinical obligations. Allocating time for sleep amidst these responsibilities often falls to a lower priority in favor of hospital work or studying for exams and rotations, which play pivotal roles in acceptance to residency. While this may weigh more heavily on students pursuing competitive residency positions, the issue of poor sleep quality and deprivation is common among all students. This dilemma presents a tragic irony: those most acutely aware of the deleterious effects of sleep deprivation on the body often find themselves without sleep in the pursuit of a career where they advise people to care for their sleep health.

A historical examination of medical education reveals the influence of figures such as William Halsted, whose reliance on stimulants to maintain efficiency, in shaping residency and medical school programs [1]. This tradition persisted until recently, with excessive workloads hindering students and residents from obtaining adequate rest. Although regulations implemented in 2003 that limited workweeks to 80 hours aimed to mitigate this issue and improve patient care outcomes, adherence remains imperfect [2].

Sleep deprivation in medical residents is often attributed to job structure, workload, and overarching medical system framework [3]. However, medical students are not relied upon as heavily in these aspects, so there may be an opportunity for change. This is especially important in the era of pass/fail Step 1 as students on rotations are trying to excel in the hospital and academically for a stronger chance at their desired specialty. Just as patients are less likely to quit smoking if their physician smokes cigarettes, the encouragement of medical students to sacrifice sleep for the sake of achievement raises questions about when a physician would counsel a patient in a similar position to improve sleep hygiene [4,5]. Despite ample evidence that such sacrifices do not yield success, the practice of sleep deprivation in medical education persists [6,7].

In medical school, both preclinical and clinical years demand a commitment to test performance and activities. However, the clinical years, dominated by clerkships that consume up to 12 hours per day, intensify the time constraints students face. These time constraints impose an opportunity cost to sleep. Consequently, third-year and fourth-year medical students may experience diminished sleep satisfaction and fewer opportunities for rest compared to their first-year counterparts. Despite this assumption, research exploring this phenomenon remains scarce.

While existing literature establishes the positive impact of sleep on test performance among medical students and its correlation with better outcomes among physicians, there is a notable gap in understanding how the transition from preclinical to clinical training affects sleep quality [8,9]. There are limited studies that have employed the validated Epworth sleepiness scale (ESS) to study sleep deficits in medical students; thus, the authors have chosen to use this measure [10,11]. The questionnaire uses a simple scale to describe the likelihood of dozing into sleep across eight common scenarios, varying in the degree of appropriateness and danger, such as while reading or driving. By comparing the ESS of students during the preclinical and clinical years of medical school, this study aims to quantitatively describe how the increased responsibilities of clinical training might impact the sleep quality of medical students.

## Materials and methods

Study population

Our survey was sent to all medical students attending the California University of Science and Medicine between April 2023-January 2024. All survey participants were contacted via their school email due to time constraints and feasibility. This is a private medical school located in Southern California with approximately 130 students per class. All first-, second-, third-, or fourth-year students who completed the survey were included. Those that did not submit a complete response for the ESS portion of the survey and duplicate responses were excluded. This study was given Institutional Review Board Approval prior to the survey being sent out.

Survey structure

All responses were collected via a Microsoft form. The survey was anonymous and voluntary. At the start of the survey, each participant created a four-digit unique identifier consisting of the last two digits of their phone numbers, as well as the day of their birth (from 01-31). The primary outcome of the survey consisted of the standard eight-item question set included in the ESS (Appendix 1). The ESS is a widely used questionnaire in the field of sleep medicine that serves as a subjective measure of an individual’s sleepiness. The rest of the survey collected demographic data and additional questions regarding sleep quality/quantity and attitudes toward medical training. In total, there were 17 questions on the survey, and responses were primarily collected via multiple choice, with the anonymous identifier, age, and total sleep time being the only free response items (Appendix 1). To provide adequate time for students to respond and to gather a reasonable sample, responses were accepted between April 2023 and January 2024 with periodic reminders sent out to all classes. Based on the unique identifiers created, any duplicate responses received were removed.

Statistical analysis

In order to best understand factors that increase the risk of excessive sleepiness, the relative risks for having an ESS greater than 10 were calculated with 95% confidence intervals and compared for various subgroups in our sample. Additionally, a univariate logistic regression analysis was carried out to assess the effect of covariates on our primary outcome and see what factors were most predictive of excessive sleepiness. In a supplemental analysis, differences in self-reported total sleep time and sleep latency were compared between subgroups using an independent two-tailed t-test. These tests were all carried out at a significance level of 0.05. All data analysis was performed using Statistical Product and Service Solutions (SPSS, version 28.0.1.0; IBM SPSS Statistics for Windows, Armonk, NY).

## Results

Demographics

In total, our sample consisted of 124 medical students with females comprising 58.9% (Table 1). This represents a response rate of 23.8% (124/520). Only 11.3% (n=14) were somewhat dissatisfied or very dissatisfied with their medical school experience.

**Table 1:** Demographics by Sleep Quality ESS = Epworth Sleepiness Scale, SD = Standard Deviation

			Sleepiness Subgroup	
		Overall (n=124)	ESS<10 (n=87)	ESS≥10 (n=37)
Sex %	Female	58.9	59.8	56.8
Age	Mean (SD)	25.4 (3.2)	25.4 (3.7)	25.3 (1.5)
Year %	M1	37.9	41.4	29.7
	M2	25.0	26.4	21.6
	M3	29.8	24.1	43.2
	M4	7.3	8.0	5.4
Currently on Clinical Rotations	% Yes	33.9	26.4	51.4

Relative risk analysis

**Figure 1:**
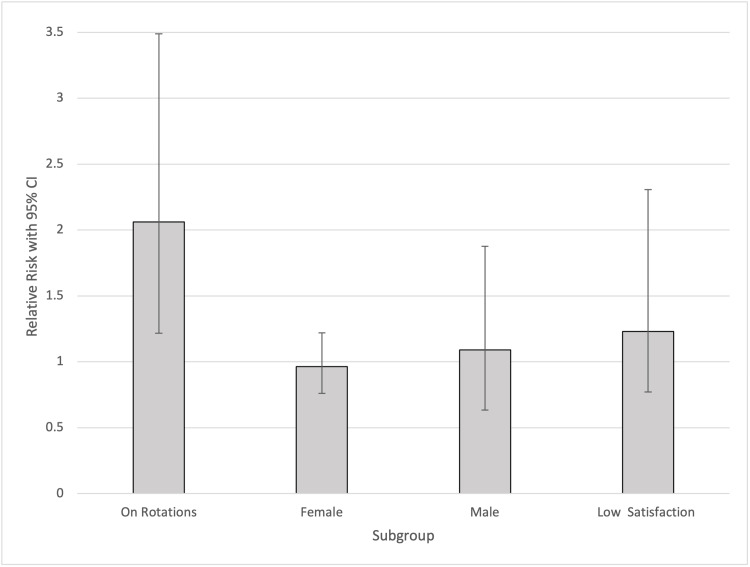
Relative Risk of ESS > 10 Between Subgroups CI = Confidence Interval, ESS = Epworth Sleepiness Scale

The relative risk of having an ESS > 10 when on clinical rotations was 2.06 (95% CI: 1.22-3.49). The relative risk of ESS > 10 was 1.09 (95% CI: 0.63-1.87) and 0.96 (95% CI: 0.76-1.22) for males and females, respectively. Finally, for students only somewhat satisfied with their medical school experience and lower, the relative risk of ESS > 10 was 1.23 (95% CI: 0.57-2.63) (Figure 1).

Logistic regression analysis

In an unadjusted logistic regression model, currently being on clinical rotations (OR: 3.2, 95% CI: 1.4-7.4) was the only significant predictor of students experiencing an ESS > 10 (Table 2).

**Table 2:** Univariate Logistic Regression Model for Predictors of ESS > 10 OR = Odds Ratio, CI = confidence interval, ESS = Epworth Sleepiness Scale

Covariate	OR (95% CI)	P-value
Currently on Rotations	3.2 (1.4 to 7.4)	0.006
Age	0.9 (0.8 to 1.1)	0.398
Gender	1.1 (0.5 to 2.4)	0.905
Low Medical School Satisfaction	1.5 (0.5 to 5.1)	0.505

Supplemental analysis

The average self-reported total sleep time for our sample was 7.17 hours (SD: 0.93) (AS1) (JM2) and 10.5% (n=13) of students had a sleep latency of over one hour. Of note, while students on rotations had a statistically significant lower total sleep time when compared to students not on rotations, mean sleep time was still roughly seven hours and is unlikely to be clinically significant. No significant difference was noted between male and female students (Table 3).

**Table 3:** Differences in Self-Reported Total Sleep Time and Sleep Latency Between Subgroups SD = Standard Deviation

Group	Total Sleep Time (Mean and SD)	P-Value	Sleep Latency (Mean and SD)	P-Value
On Rotations	6.93 (0.78)	0.040	0.79 (0.50)	0.830
Not on Rotations	7.29 (0.98)		0.81 (0.46)	
Males	7.05 (0.70)	0.235	0.78 (0.46)	0.637
Female	7.25 (1.06)		0.82 (0.48)	

## Discussion

The data gained from this pilot study suggest that the use of the ESS to evaluate excessive daytime sleepiness (EDS) may be feasible in a medical student population. Our data suggest that the risk of medical students experiencing EDS, defined by an ESS > 10, doubles when students are on clinical rotations. While our study recorded roughly 30% of students reporting an ESS > 10, similar studies have noted a prevalence of EDS with ESS > 10, ranging from 25% and 60% among medical students compared to a population prevalence of 19.1% [8,12-14]. Johnson et al. reported that 25% of second-year medical students surveyed had an ESS > 10 and that those with < 6 hours of sleep were four times more likely than those with ≥ 7 hours to experience nodding off while driving [12]. This impairment extends beyond driving, and examinations of medical students found that sleep deprivation of > 2 hours resulted in significantly lower concentration scores even in students who had not worked a night shift. This decline was more prominent in students who had > 2 hours of sleep deprivation and had worked a night shift [15]. Poor sleep quality may also impair academic performance as evidenced by multiple studies; however, Johnson et al. failed to show this connection in their cohort [12,14,16,17].

One likely factor for the higher prevalence of EDS in students on clinical rotations is the introduction of the night shift. Two critical determinants of sleep quality and quantity are the timing of sleep opportunity and work. Though students are afforded a day to transition, the sudden shift to daytime sleeping may not provide restful sleep. Other students may opt to retain the normal schedule in favor of an extended period of wakefulness [18]. Additionally, studies examining night-shift workers have shown that workers are unlikely to show adequate changes in melatonin or cortisol rhythms within three consecutive night shifts [19]. Due to the rapid pace of learning and work demands, students are scheduled into shifts when their bodies will not have enough time to adapt. This biochemical dysregulation and sudden increase in sleep scarcity may decrease sleep quality, which may explain why our data show clinical rotations as the only significant predictor of student ESS > 10 despite those on rotations still sleeping roughly seven hours a night. Sleep latency did not play a significant role in having an ESS > 10 among our cohort.

This study comes with many limitations. First, ESS is generally used for sleep apnea and has had its test-retest reliability called into question. However, this does not refer to its utility focusing on comparing group scores and has shown consistent results in healthy young subjects [13,20]. Second, we did not distinguish which rotation third-year medical students were on which may affect the results if a disproportionate number of respondents were on demanding rotations. Additionally, due to the sampling method used in this study, many different forms of information bias may have been introduced into our results. For one, recall bias may be present since sleep deprivation can be intermittent and respondents who were currently asymptomatic may not recall their symptoms and vice versa. Furthermore, the use of a Microsoft form for survey purposes may lead to performance bias as medical students are more aware of sleep issues in their field, which may skew their responses. Finally, due to our relatively small sample size from a single institution and non-random recruitment, our results are likely subject to selection bias and may not be completely generalizable or reproducible with medical students across the country, especially given our response rate of 23.8%.

Despite these limitations, it is still worth noting that our results are consistent with evidence built in previous literature and may point out a valuable area of study for further work [14,21]. Future studies exploring the impact of EDS on students could take a multi-institutional approach and examine the differences between students on rotations at a tertiary, community, or rural hospital. Further stratification of students by which clinical rotation they are on and how recently they worked a night shift at the time of data collection may help narrow down causes. A qualitative approach examining what activities students spend time on outside of the hospital and their prospective specialty may also provide context for datasets such as this one. Additionally, comparing average shelf scores combined with average sleep quality and the most recent rotation may offer insights into how demanding rotations and poorer sleep quality impact academic performance. Finally, systematic, random sampling methods are needed to best capture the relationship of interest without the introduction of bias.

## Conclusions

The demands of medical school are rigorous and grow as students progress; however, the marked increase in EDS upon beginning clinical rotations stands out amongst other years and is worthy of further investigation. The current study finds that a medical student’s risk of experiencing EDS while on clinical rotations may double when compared to their pre-clinical counterparts. While EDS is certainly multifactorial, evidence suggests that night shifts and the consequent sleep dysregulation may play a pivotal role. Despite its limitations, this study provides interesting pilot data on the quantitative examination of sleepiness among medical students and may be used to guide areas for future work.

## Appendices

Appendix 1. Survey questions

1. Please create a 4-digit anonymous identifier by entering the last 2 digits of your phone number followed by the two digits of the day you were born (if you were born between the first 9 days of the month, please enter it with a 0 in front. eg. 01 for the 1st of a month).

2. What year of medical school are you in?

3. Are you currently on clinical rotations?

4. How old are you? (please answer with whole numbers)

5. Gender

Items 6-14 were rated with the following four response options:

0 = Would never nod off

1 = Slight chance of nodding off

2 = Moderate chance of nodding off

3 = High chance of nodding off

6. Sitting and reading

7. Watching TV

9. Sitting, inactive, in a public place (e.g., in a meeting, theater or dinner event)

10. As a passenger in a car for an hour or more without stopping for a break

11. Lying down to rest when circumstances permit

12. Sitting and talking to someone

13. Sitting quietly after a meal without alcohol

14. In a car, while stopped for a few minutes in traffic or at a light

15. How many hours of sleep do you get in a night? (please answer with your best whole number estimate)

16. How long does it take you to fall asleep once in bed? 

A) Under 30 minutes B) Under 1 hour C) Under 2 hours D) More than 2 hours

17. How satisfied are you with your experience in medical school?

A) Very Satisfied B) Somewhat Satisfied C) Neither Satisfied nor Dissatisfied D) Somewhat Dissatisfied E) Very Dissatisfied
